# The Impact of Mobile-Assisted Language Learning on English as a Foreign Language Learners’ Vocabulary Learning Attitudes and Self-Regulatory Capacity

**DOI:** 10.3389/fpsyg.2022.872922

**Published:** 2022-06-21

**Authors:** Xiao Lei, Jalil Fathi, Shabnam Noorbakhsh, Masoud Rahimi

**Affiliations:** ^1^Department of Foreign Languages, Zunyi Medical University, Zunyi, China; ^2^Department of English and Linguistics, Faculty of Language and Literature, University of Kurdistan, Sanandaj, Iran; ^3^Department of English and Linguistics, Faculty of Language and Literature, Islamic Azad University, Sanandaj Branch, Sanandaj, Iran

**Keywords:** mobile-assisted language learning, vocabulary learning attitudes, self-regulatory capacity, EFL, latent curve modelling

## Abstract

Over the past decades, English as a foreign language (EFL) learning has witnessed a heightened interest in the role of mobile-assisted language learning (MALL) in vocabulary learning. To shed more light on the impact of MALL on vocabulary learning, this study, employing a quantitative longitudinal design, aimed at examining the impact of a MALL programme on 139 EFL learners’ vocabulary learning attitudes and self-regulatory capacity. To this end, this study investigated the latent change score models of the learners’ vocabulary learning attitudes and self-regulatory capacity over time. Over the course of 1 year, various mobile applications were integrated into the regular English language instruction of the learners. The required data were collected *via* administering vocabulary learning attitude and self-regulating capacity in vocabulary learning scales. The data were analysed applying latent growth curve modelling to examine the participants’ longitudinal trajectories and patterns of change in the two waves of collected data. The fit indices of the latent change models revealed an increase in both the EFL learners’ vocabulary learning attitudes and their self-regulatory capacity during the 1-year MALL programme. The analysis of between-person differences also indicated that changes in both variables were positively correlated.

## Introduction

As one of the viable and alluring technological devices, mobile devices have become widely available and advocated in educational settings (Looi et al., 2010; Wang et al., 2017; Hwang and Fu, 2018; Liu et al., 2018; Rassaei, 2019; Fathi et al., 2021). Mobile learning refers to the use of mobile technologies, such as smartphones and tablets (Huang et al., 2016), for educational purposes. Mobile-based learning is considered “learning across multiple contexts, through social and content interactions, using personal electronic devices” (Crompton, 2013, p. 4). In contrast to conventional computer-based learning technologies, which are constrained by time and place, mobile-based technologies provide their users with ubiquitous access that is not constrained by spatial and temporal factors (Kukulska-Hulme and Traxler, 2005; Melhuish and Fallon, 2010; Wong, 2012; Wang and Shih, 2015). That is, the mobility of mobile-based technologies, like mobile phones, iPod, and tablets, provide second language (L2) learners with a convenient place and time and great learning opportunities (Sharples, 2006; Laurillard, 2007), which might not be supplied by desktop computers (Chinnery, 2006).

In line with mainstream education, the use of mobiles in language learning, or what is currently known as mobile-assisted language learning (MALL), gained much popularity (Kiernan and Aizawa, 2004; Hwang and Fu, 2018), as it was included in the foreign language curriculum and offering new learning devices to the net generation (Oblinger and Oblinger, 2005). MALL has received much interest because of its authentic and contextual language learning experiences (Chinnery, 2006; Kukulska-Hulme, 2006; Shadiev et al., 2017; Cheng and Chen, 2022). Previous research on MALL has examined the effect of mobile-based technologies on EFL learners’ language learning skills, such as speaking (Sun et al., 2017), writing (Eubanks et al., 2018), listening (Jia and Hew, 2019), and reading (Chang and Hsu, 2011), and language sub-skills, such as grammar (Chu et al., 2019), vocabulary (Çakmak and Erçetin, 2018), and pronunciation (Wongsuriya, 2020). In comparison with all the MALL-related studies, the effect of mobile devices on vocabulary learning has attracted the most attention (Burston, 2014; Duman et al., 2015).

Although the impact of mobile-based technologies on English language vocabulary learning has been explored extensively (Stockwell, 2010; Çetinkaya and Sütçü, 2018; Lin and Lin, 2019), no large-scale studies have been carried out so far to check the potential impact of MALL on vocabulary learning attitudes in an EFL context. Following Masgoret and Gardner (2003), vocabulary learning attitude could be conceptualised as learners’ positive/negative reactions towards their vocabulary learning. Positive attitudes towards second/foreign language learning (in this study, positive attitudes towards vocabulary learning) enhance learners’ second/foreign language learning motivation and help them easily accomplish their language learning tasks (Ellis, 2008; Cheung and Hew, 2009; Nation and Webb, 2011; Burston, 2015; Chen et al., 2020; Soyoof et al., 2021, 2022). Therefore, positive attitudes towards vocabulary learning can facilitate second/foreign language learners’ vocabulary learning.

The present study, therefore, is aimed to examine the influence of a 1-year MALL programme on EFL learners’ vocabulary learning attitudes. That is, a quantitative longitudinal research design is adopted in this study to investigate EFL learners’ longitudinal trajectories and patterns of change in vocabulary learning. In addition, as the concept of self-regulation (Zimmerman and Schunk, 2008), which refers to learners’ activating and sustaining planned self-generated thoughts, emotions, and activities to achive their learning goals (Zimmerman, 2000, 2008), has received considerable attention in the MALL context (e.g., García Botero et al., 2021), especially in mobile-based vocabulary learning context (e.g., Barth et al., 2020). EFL learners’ self-regulatory vocabulary learning changes over the 1-year MALL programme is also examined in the present study. Moreover, the reciprocity and relationship between EFL learners’ vocabulary learning attitudes and their self-regulatory vocabulary learning are checked in this study.

As Engeström’s (1987) activity theory, which was adopted in the current study, explores learners’ gradual movement from other-regulation (i.e., interactive activities among peers) to self-regulation (i.e., learners’ autonomous functioning), we investigated the EFL learners’ self-regulatory vocabulary learning to check if EFL learners reach their self-regulation in vocabulary lrarning. In addition, as collaborative activities among EFL learners occur infrequently in the current study on the one hand, and EFL learners rarely use mobile-based applications for their English language courses, exploring the EFL learners’ vocabulary learning attitudes in this new learning environment could shed more light on the related literature. Thefindings of the study can add to the literature given that no quantitative longitudinal growth modelling analysis has been carried out examining learners’ vocabulary learning attitudes and self-regulation in the EFL context. The findings of the present study can also propose practical implications to contribute to EFL learners’ vocabulary learning attitudes and their self-regulatory vocabulary learning in a mobile-based vocabulary learning context.

## Literature Review

The theoretical framework of the present study follows Engeström’s (1987) activity theory. This theory originates from Vygotsky’s (1978) social constructivism in which learners’ self-regulation (i.e., internalisation of different skills) precedes their other-regulation (i.e., interactions and mediations among peers). Vygotsky’s (1978) conceptualisation of learning comprises three components of subject (e.g., EFL learners and teachers), object or the desired goals that the learners want to achieve by the activity (e.g., improvements in vocabulary learning), and the mediational tools or artefacts (e.g., online applications) that mediate between the subject and object (see, for instance; Ebadi and Rahimi, 2019; Fathi and Rahimi, 2020; Rahimi and Fathi, 2021). However, activity theory conceptualises learning as interactions among the subject, object, and artefact, rules or norms (e.g., using mobile devices), community to which learners share a common goal (e.g., class and institution), and division of labor which refers to learners’ responsibilities in accomplishing an activity in the community (e.g., instructors teaching EFL vocabulary and learners learning EFL vocabulary; Engeström, 2001). Based on this theory, learning could be regarded as an activity system in which learners are mediated by the tools to construct knowledge. Overtime, learners are expected to internalise the knowledge and act autonomously without receiving mediations from the other capable individuals and achieve their self-regulation.

The conceptualisation of activity theory is similar to the Vygotskyan constructivist theory used in Ebadi and Rahimi (2017, 2018, 2019), Rassaei’s (2020), and Rahimi and Fathi (2021) study, as students are involved in collaborative activities to provide and receive both explicit and implicit mediations. In such collaborative activities, students gradually provide more explicit mediations on their peers’ tasks only if the peers are not able to address their language issues through more implicit mediations. Students who address their language issues through minimal or implicit peer mediations are believed to be closer to their self-regulation in learning. However, activity theory goes beyond the Vygotsky’s social constructivist theory by considering not only the students, their peer mediations, and their language learning goals, but also the rules or norms that the students are required to consider, like applying special language learning tools, the context where the students are engaged in the collaborative language learning activities, such as classroom context, and the students’ responsibilities to do the target activity, like learning EFL vocabulary. That is, activity theory considers a wider range of factors while specifically focusing on students’ collaborative learning activites.

Activity theory fits well with MALL desin and, in particular, with mobile-based vocabulary learning, since it can “capture the dynamic nature of activity systems and changing points of focus over time” (Levy and Stockwell, 2006, p. 119). On the other hand, as mobile-based learning is an activity that happens in the physical and social environments (i.e., interactions among learners and the community, rules, and norms of the activities) of learning, it corresponds to the principles of activity theory (Wali et al., 2008). Moreover, a number of studies have adopted activity theory in mobile-based language learning environment (e.g., Uden, 2007; Liaw et al., 2010).

Following this theory, the EFL learners in the present study are mediated by their peers and the researcher/instructor to accomplish the vocabulary-based activities in the mobile learning environment. The learners, considered the subjects in the activity theory, form the learning community, since they all follow a common goal (i.e., improving their vocabulary knowledge) by sharing the responsibilities among the members to accomplish vocabulary-based activities using mobile applications. In the current study, activity theory helped explore the interactions between learners and their social and cultural contexts comprehensively, identify the constraints in the EFL MALL context, and capture longitudinal changes (e.g., Ellis, 2015; Soyoof et al., 2021) in EFL vacobulary learning attitudes and self-regulatory capacity in vocabulary learning. Activity theory has not been adopted in similar studies; as a result, the current study is believed to shed light on the literature.

### Mobile-Assisted Vocabulary Learning

Vocabulary learning, considered a prominent factor in English language learning (Nation, 2001, 2020; Zou et al., 2021), might be facilitated through mobile-assisted technologies (Hulstijn and Laufer, 2001). Following Sung et al. (2016), the user-friendliness of mobile-based technologies allows L2 learners to spend their free time learning new vocabulary items. In addition, applying mobile-assisted technologies, learners can learn at their own pace (Norris et al., 2011; Hung et al., 2012). For instance, some mobile vocabulary learning applications allow L2 learners to download different types of content for offline study, others supply context-appropriate words by activating Global Positioning System (GPS) to identify learners’ locations (Godwin-Jones, 2011).

A substantial body of studies have employed mobile phones for L2 vocabulary learning (Levy and Kennedy, 2005; Stockwell, 2010; Lai, 2016; Xu and Peng, 2017; Çetinkaya and Sütçü, 2018; Rosell-Aguilar, 2018; Lin and Lin, 2019; Seibert Hanson and Brown, 2020). Studies in this regard have specifically focused on the effect of short messages (Kennedy and Levy, 2008), electronic dictionaries (Song and Fox, 2008) and flashcards (Başoğlu and Akdemir, 2010). Lai (2016), for instance, applied WhatsApp (a mobile instant messenger) to provide a mobile immersion context in order to explore Chinese EFL students’ vocabulary learning through an experimental-control group design. A number of high-frequency English verbs were introduced to the participants each session and the students were supposed to get engaged in some relevant text-chat discussions. Although the findings revealed no significant difference between the mobile-based and control groups’ vocabulary learning after 3 months of 1.5-h weekly meetings, significant correlations were observed between the students’ chat frequency and vocabulary gains in the mobile-based group. Lai also indicated that students’ mentality towards the interactive learning environment in smartphone might facilitate or hamper the effectiveness of the mobile immersion.

Lin and Yu (2017) examined Taiwanese EFL learners’ vocabulary learning through mobile phones. The participants of the study were supposed to learn four groups of target words each of which presented either in text, text and picture, text and sound, or text, picture, and sound mode in four different weeks. Having conducted a vocabulary test and a questionnaire related to cognitive load at the end of the programme, the findings indicated the significant role of audio input in recalling new word meanings and reducing the cognitive load of learning the new words. The findings also revealed the EFL learners’ positive attitudes towards the mobile assisted vocabulary learning programme.

Wu (2015) also investigated the impact of smartphone application on EFL learners’ vocabulary learning through an experimental research design. In the experimental group, the students covered the material through some mobile-based applications, while in the control group, the students covered the same lessons in print. The findings indicated that the students in the mobile-supported class outperformed those in the control group on their English vocabulary learning. Thornton and Houser (2005) further explored the effect of a mobile-based technology classroom on Japanese EFL students’ vocabulary learning through a survey. The results showed that the students using mobile devices received higher vocabulary learning marks in comparison with the students learning on websites and paper. The students in the mobile-based technology classroom also had positive attitudes towards learning English idioms through mobile devices.

Similarly, Li and Hafner (2022) examined EFL learners’ vocabulary learning through mobile-assisted word cards and paper word cards. They applied an experimental-control group research design to check the learners’ word knowledge in two areas of receptive knowledge of form-meaning connection (i.e., comprehending words in listening and reading) and productive knowledge of collocations (i.e., producing words in speaking and writing; Schmitt, 2010). The findings indicated that both the mobile-assisted and conventional classes improved the learners’ vocabulary knowledge; however, the mobile-assisted class outperformed the conventional class in this regard. In addition, Liu (2016) exploring the effect of instant message function of smartphones on EFL learners’ vocabulary knowledge and vocabulary retention, found that instant message group improved their vocabulary knowledge and retention and outperformed their conventional group counterpart. The results of the delayed post-tests further indicated that mobile-assisted learning was effective in long term provided that learning strategies be applied effectively.

In a similar vein, Rachels and Rockinson-Szapkiw (2018) applied a Duolingo-based instructional game course to develop L2 learners’ vocabulary knowledge in a gamified learning environment. In comparison with a conventional instruction, Duolingo-based instruction greatly enhanced the learners’ motivation while it had subtle impact on the learners’ vocabulary learning. Loewen et al. (2019) also revealed language learners’ improvement on Duolingo at the end of a term. Loewen et al. further showed a positive relationship between the amount of time spent on Duolingo and learning achievements. The participating learners also had positive perceptions towards the flexibility and gamification aspects of Duolingo. Lu (2008) and Zhang et al. (2011), respectively, exploring Taiwanese and Chinese EFL learners’ vocabulary learning through mobile-assisted technologies, found that mobile-supported groups’ vocabulary learning was improved and these improvements outperformed those of the conventional group; however, in Zhang et al. (2011) study, the vocabulary learning improvements did not last in the delayed post-test.

Additionally, Wong and Looi (2010) explored EFL students’ English language preposition learning through two MALL case studies. The students using mobile devices constructed English sentences through some newly acquired prepositions. The students were consequently engaged in online or classroom discussion to develop their understanding of the English language prepositions. Finally, Kurt and Bensen (2017) adopted Vine (i.e., a mobile application to record and shar videos) to help EFL learners develop their vocabulary learning by speaking and spelling through the mobile devices and review their knowledge by the video clips. The findings revealed that Vine-based practice improved the learners’ vocabulary knowledge and enhanced their motivation for learning vocabulary.

### Self-Regulation in Vocabulary Learning

Self-regulation could be conceptualised as “the process by which learners personally activate and sustain cognitions, affects and behaviours that are systematically oriented towards the attainment of learning goals” (Zimmerman and Schunk, 2008, p. vii). Having originated in Corno and Mandinach’s (1983) theory, self-regulation comprises three main components of strategic actions, which comprises planning, monitoring, and evaluating strategies, learning motivation, which focuses on learner engagement, and self-efficacy, which focuses on learner abilities to achieve a goal (Chen and Hsu, 2020).

In spite of a significant body of research pertaining to vocabulary learning strategies (Tseng and Schmitt, 2008; Chacón-Beltrán, 2018; Wyra and Lawson, 2018; Schmitt and Schmitt, 2020; Teng, 2020), there have been various conceptualisations regarding the concept of learning strategy (Dörnyei, 2005; Tseng et al., 2006; Schmitt and Schmitt, 2020). As a different perspective towards vocabulary learning strategies, Tseng et al. (2006) regarded strategic vocabulary learning as learners’ self-regulation in language learning and developed a questionnaire to examine learners’ self-regulatory capacity in vocabulary learning. Tseng et al. questionnaire incorporates the concept of self-regulation into the field of L2 learning and operationalises learning strategies as self-regulatory capacity. In other words, it is argued that strategic learning has been reconceptualised through the theoretical lens of self-regulated learning. However, not many empirical studies have investigated the concept of self-regulating capacity in vocabulary learning, especially in EFL settings.

According to Azevedo and Cromley (2004), learners with high self-regulation skills outperform those with low self-regulation skills when it comes to technology-enhanced learning space. On the other hand, technology-enhanced learning tools facilitate such self-regulation skills as goal setting, task-based strategies, time management, collaborative learning activities, and self-evaluation (Carneiro et al., 2007; Shea and Bidjerano, 2010; Lai and Gu, 2011; Fathi et al., 2018; Zheng et al., 2018). Following Chen et al. (2008) and Sha et al. (2012), self-regulated learning skills and mobile-based technologies are closely interrelated and promoting either of them can heavily influence the other one. It is also argued that the flexibility of mobile-based technologies in delivering content and increasing motivation and engagement can positively affect students’ self-regulation (Sung et al., 2015, 2016) which can consequently raise their self-awareness, self-study behaviour (Kondo et al., 2012), and positive attitudes (Zimmerman and Schunk, 2008; Ning and Downing, 2010).

The concept of self-regulation is an under-researched concept in the MALL context (Sha et al., 2012; Hernández and Rankin, 2015; García Botero et al., 2021). In the EFL context, Chen et al. (2019), for instance, explored Taiwanese EFL learners’ vocabulary learning along with a self-regulation mechanism through a mobile-assisted application. Adopting an experimental-control group design, the learners in one group received an English vocabulary-based application with a self-regulation mechanism while the other group received English vocabulary learning application without a self-regulation mechanism. The findings showed that learners receiving self-regulated learning had better learning performance and motivation.

In a similar vein, Barth et al. (2020) exploring Chinese EFL students’ attitudes and perceptions towards design factors supporting self-directed mobile-based vocabulary learning, found that scaffolding in the form of first language translation developed basic and high-frequency vocabulary learning in mobile learning environments. The findings further propose that in order to move mobile learning to mainstream education, students’ capacity for self-directed learning need to be enhanced beforehand. Haq (2019) also indicated that MALL positively influenced EFL learners’ self-regulated learning strategies. Haq maintained that there were no differences between self-regulated learning strategies of EFL learners with different proficiency levels. Additionally, García Botero et al. (2021) exploring L2 students’ self-regulation in a mobile-based environment using Duolingo, revealed that students being trained in self-regulation showed a higher participation in Duolingo and higher test marks in L2 writing.

## Purpose of the Study

As the literature review revealed, mobile-based applications had positive effects on EFL vocabulary learning attitudes (Wu, 2015; Kurt and Bensen, 2017; Lin and Yu, 2017) and self-regulatory capacity in EFL vocabulary learning (Chen et al., 2008; Sha et al., 2012; Haq, 2019). However, vocabulary learning attitudes and self-regulatory capacity in vocabulary learning have not been explored in quantitative longitudinal research designs in EFL context (Zhang and Zou, 2020). Given the fact that L2 learning takes a long time, comprehensive understanding of L2 processes and changes should be investigated over time (Ortega and Byrnes, 2009). Therefore, we employed a quantitative longitudinal research design and adopted Engeström’s (1987) activity theory to examine the EFL learners’ longitudinal trajectories and patterns of change for vocabulary learning attitudes and self-regulatory capacity in vocabulary learning in a 1-year MALL programme. The reciprocity and relationship between these two factors were also examined. Employing quantitative longitudinal research designs have “several conceptual, methodological, and practical advantages and can stimulate the development and empirical examination of more complex questions and models concerning L2 development over time” (Barkaoui, 2014, p. 65). To accomplish the purposes of the study, the following research questions are formulated:

1. What are the effects of a 1-year MALL programme on EFL learners’ vocabulary learning attitudes and self-regulatory vocabulary learning?
2. What are the relationships between EFL learners’ vocabulary learning attitudes and self-regulatory vocabulary learning?

## Materials and Methods

### Design of the Study

The current study adopted a longitudinal research design to collect quantitative data about EFL learners’ vocabulary learning attitudes and self-regulatory vocabulary learning in a 1-year MALL programme. Caruana et al. (2015) defined longitudinal studies as continuous or repeated investigations of learners over a long time without any external influences being applied. Longitudinal research is believed to provide the researchers with rich data, since it investigates the participants in a long period of time.

### Participants

The participants recruited for the purpose of the present study were 175 EFL learners (78 males, and 72 females) in the age range of 16–40, studying in a private English language institute in Kurdistan, Iran. Convenience sampling method (Dörnyei, 2007) was applied to choose the participating learners in the present study. That is, sample selection followed the researcher/instructor’s certain practical criteria, like the participants’ “geographical proximity,” their “availability at a certain time,” their “easy accessibility,” and their “willingness to volunteer” in the current study (Dörnyei, 2007, p. 99). As the researcher was teaching at that private language institute, selecting the learners, who were studying at the language institute, was convenient for the researcher. However, at the beginning 4 months of the study, 36 participants dropped out of the study due to being reluctant and/or unmotivated to continue the intervention. Thus, 139 EFL learners, as the total number of participants of this quantitative longitudinal study, underwent a 1-year MALL programme.

The participants were not at the same English language proficiency level. Before embarking upon the study, all the learners had gone through 4–7 years of English learning course in public school; however, some of the participants had experienced more than 7 years due to the additional university education. As a result, all the learners took full-course placement test which was designed and validated by the authors. Based on the test results, the learners were assigned to beginner, intermediate, or advanced level to cover different coursebooks of Top Notch Fundamentals, 1, 2, 3, and Summit 1 and 2. Except for five participants, the rest (*N* = 134) had computer literacy and they all had access to smart phones. The information about the participants is provided in more detail in Table 1.

**Table 1:** Demographic information of the participants.

		Frequency	Percentage
Gender	Female	61	43.8
	Male	78	56.9
	Total	139	100.0
Age	16–25	103	74.1
	Above 25	36	25.8
	Total	139	100.0
Computer literacy	Yes	134	96.4
	No	5	3.5
	Total	139	100.0
Smart phone availability	Yes	139	100.0
	No	0	0.0
	Total	139	100.0
Multilingualism	Kurdish	137	98.5
	Farsi	0	0.0
	Kurdish-Farsi	2	1.4
	Total	139	100.0

### Instruments

#### Vocabulary Learning Attitude Scale

In order to measure the vocabulary learning attitudes of the participants in the current study, vocabulary learning attitude scale (VLAS) developed by Tseng and Schmitt (2008) was employed. The scale comprises 10 items, each targeting the attitude of participants towards their vocabulary retrieval. It is a six-point Likert scale that range from 1 = “strongly agree” to 6 = “strongly disagree” in 10–60 score range. The validity of VLAS was found to be acceptable. Additionally, the internal consistency of the scale, as measured by Cronbach’s Alpha formula, was reported to be 0.86 in this study.

#### Self-Regulating Capacity in Vocabulary Learning Scale

To measure the EFL learners’ self-regulatory capacity in vocabulary learning, self-regulating capacity in vocabulary learning scale (SRCV), which was developed and validated by Tseng et al. (2006), was employed in the present study. SRCV includes 20 items with six-point Likert scale which each ranges from 1 = “strongly agree” to 6 = “strongly disagree”; the scale is in 20–120 score range. This instrument addresses five subscales of commitment control (i.e., setting goals for vocabulary learning); metacognitive control (i.e., keeping concentration on vocabulary learning); satiation control (i.e., overcoming boredom in vocabulary learning); emotion control (i.e., controlling stress in vocabulary learning); and environment control (i.e., dealing with contextual factors effectively). The validity of SRCV was checked by Tseng et al. (2006) and the results were acceptable. In addition, the internal consistency of SRCV (i.e., the reliability of SRCV) was measured through Cronbach’s Alpha the present researchers and the outcome was 0.85, which was also acceptable.

### Procedure

The intervention started at the end of June, 2015 and lasted at the end of June, 2016. The main coursebooks were *Top Notch and Summit series* which were required to be covered during the 1-year intervention. To homogenise different groups of learners and place them at their right level of language proficiency, all the participants were required to take a full-course placement test. The results were interpreted following the standards of the language institutes as A1 for Top Notch Fundamentals and Top Notch 1, A2 for Top Notch 2, B1 for Top Notch 3, B2 for Summit 1, and C1 for Summit 2. The participants were then provided with one of Top Notch coursebooks appropriate to their proficiency level. Prior to the treatment, SRCV and VLAS were administered by the researcher/instructor to specify the learners’ initial level of self-regulation and attitudes towards EFL vocabulary learning. Next, a set of multiple mobile applications were integrated into the 1-year MALL programme. At the end of the 1-year MALL programme, SRCV and VLAS were re-administered to collect the second wave of the data.

#### The Applications Used in the MALL Programme

The list of the language learning applications and their descriptions employed in the MALL programme is summarised in Table 2.

**Table 2:** The list of language learning applications and their descriptions.

Name of applications	Description
Merriam-Webster and Longman e-dictionaries	Provide various definitions of a term Provide collocations and idioms Provide British and American pronunciations Faster, easier, and better usage and environment
Widget of 504 and 1,100 Essential Words G5	Provides vocabularies used in sentences with Persian meaning Provides synonyms and antonyms Reminds automatically to review vocabularies daily
English songs	Improves listening with authentic songs Provides the meaning of the songs Evaluates learners’ understanding and listening with quizzes Attractive environment for better confirmation of covered vocabularies and structures
Social network applications	Provide an interactive atmosphere to speak Assimilate synchronous learning Assimilate asynchronous learning Games and Puzzles
Beetalk and call recorder	Offer a collaborative room to share ideas Allow learners to record their voice
Vocabulary for high school students	Provides a picture related to the meaning of vocabularies Provides pronunciation and synonyms and parts of speech of vocabularies
Encarta student premium	Provides audio and video files Gives a dictionary of definitions and thesauruses Provides lots of authentic materials

The selection of the mobile-based applications presented in Table 2 was due to their convenient accessibility, their free charge, the researcher/instructor’s familiarity and previous experience of using such language learning and mobile-based applications, and popularity of these applications among the learners. The following sections explain and exemplify the mobile-based applications used in the current study.

##### Merriam-Webster and Longman e-Dictionaries

First, the researcher/instructor decided to apply various dictionary applications including Longman Dictionary of Contemporary English and Merriam Webster Dictionary for the learners of all levels in order to allow them to use the dictionaries for different purposes. The researcher/instructor explained how to install the applications and how to look up the words in the dictionaries *via* their mobiles. That is, the learners were taught how to use the dictionaries for word definitions, pronunciations, part of speech, idioms, and collocations. The learners were also allowed to use these applications freely inside the class whenever they deemed them necessary. Upon applying the words accurately, the researcher/instructor could make sure that the learners regularly used the dictionaries in and outside the classroom. In addition, the researcher/instructor regularly monitored the learners while using the dictionaries.

##### Widget of 504 and 1,100 Essential Words G5

In order to improve the vocabulary knowledge of participants, the researcher/instructor also provided the learners with different Widgets of vocabulary expanding applications including *400 Essential Words* for A1 and A2 levels, *504 Absolutely Essential Words* for B1 and B2 levels, and *1,100 Words* for C1 level learners. By regularly monitoring and asking the learners about the target words, the researcher/instructor was assured that the learners regularly checked these vocabularly applications in and outside the classroom.

##### English Songs

To make the learning more interesting, the learners at all levels were introduced to *English Songs* application, enjoying three different levels appropriate for elementary, intermediate, and advanced EFL learners. Each level includes 20 songs allowing learners to learn more vocabulary in the context of songs accompanied by having access to the lyrics with Persian translation provided under each line. The learners could also access to the list of vocabulary derived directly from the songs. The researcher/instructor frequently checked the learners’ vocabularly knowledge.

##### Social Network Applications

To make the courses more communicative, the researcher/instructor decided to provide the participants with social network applications, such as *Telegram* and *WhatsApp*. In other words, the main purpose of such applications was to make the EFL learners much more engaged in their vocabulary learning. Therefore, the researcher/instructor administered some groups in Telegram through which the learners could chat together. The learners were involved in different discussions by asking and answering questions. The topics were specified either by the researcher/instructor or by the learners themselves. Taking part in English discussions in theses websites was welcomed and encouraged. The group members were told to feel free to send their messages, ask their questions, post pictures, send English quotations, and even share *Word of the Day* from their e-dictionary applications. The researcher/instructor was the manager and one of the members of these groups; as a result, different activities of the learners were monitored and checked.

##### Beetalk and Call Recorder

The learners were also exposed to *Beetalk*, which is a social network application, as well as a *Call Recorder* to record their voice while they had to make a phone conversation. They were all supposed to conduct a phone conversation according to the samples provided in their books and share the recorded files through *Beetalk* to get feedback from the researcher/instructor and their classmates. Each learner made a number of phone conversations, recorded them, and then shared the recorded voices with other learners throug Beetalk which allowed the learners to have more discussions about the recorded voices, especially about the use of vocabulary.

Afterwards, the learners were provided with MP3 audio files and audiobooks. There were numerous benefits for mobile phone-based audiobooks in comparison with their CD-ROM or cassette counterparts. They displayed a rich auditory context for the learners with different proficiency levels and in various contexts. The learners were encouraged to listen to the audio files through their mobile phones in different situations. Since they all had access to the pdf files of all stories on the website, they could enjoy using them whenever they found the MP3 stories unclear. For the purpose of the present study, the researcher/instructor introduced the learners to four-minute mp3 stories downloaded from www.americanstoryteller.com. By regularly asking the learners about the vocabulary and content of the stories and having further discussions in the classroom, the researcher/instructor ensured that the learners listened to the audios outside the classroom and understood the vocabulary and content as well.

##### Vocabulary for High School Students

As a part of the programme, *Vocabulary for High School Students* application was also provided to all the participants. It is rich in terms of having various features and providing an appropriate atmosphere for vocabulary learning. It facilitates learning vocabulary by main ideas, Anglo-Saxon, Latin, and Greek prefixes, roots, and other word elements and providing the EFL learners with accurate pronunciation, English definition and synonyms, and parts of speech, all of which were presented in either pictorial or auditory mode. The researcher/instructor made sure that the learners regularly checked *Vocabulary for High School Students* application in and outside the classroom through monitoring and asking the learners about the words.

##### Encarta Student Premium

Finally, to enrich the learners’ vocabulary knowledge by presenting authentic materials, the researcher/instructor decided to install *Encarta Microsoft Student Encyclopedia*, which includes templates and tutorials to help learners do their assignments in Microsoft Office. Microsoft Office also includes Encarta Premium which provides encyclopaedia articles, photos and illustrations, videos and animations, audios, maps, and website addresses. Encarta Dictionary is another useful feature of Encarta Premium as it provides thesaurus to find synonyms and antonyms, translations to translate a word or phrase into another language, as well as verb conjugation to conjugate verbs. Each session, the researcher/instructor specified different tasks for learners to accomplish using *Encarta Microsoft Student Encyclopedia*. In the following session, the researcher/instructor asked the learners about different vocabulary and content of the application to substantiate that the learners applied the application properly.

### Data Analysis

Following McArdle (2001) and Ferrer and McArdle (2010), we employed latent change score models and used the programme Mplus 7.11, introduced by Muthén and Muthén (2012), to measure changes. Based on this model, change is conceptualised as defining the variables at the second time wave and compare them with the sum of the score at the first time wave (McArdle and Prindle, 2008). Based on this model, change in one variable can be related to change in another variable (McArdle and Hamagami, 2001). To address the purpose of this study, we utilised the bivariate change models which include auto-regression paths in addition to crossed regression paths from the variables (i.e., vocabulary learning attitudes and self-regulatory capacity in vocabulary learning) at Time Wave 1 to the change score variables, the correlation of vocabulary learning attitudes and self-regulatory capacity in vocabulary learning at Time Wave 2, and the correlation of change scores.

To assess goodness-of-fit of the models and the *χ*^2^ value, root mean square error of approximation (RMSEA), standardised root mean square residual (SRMR), and comparative fit index (CFI) were checked. Following Hu and Bentler (1999), the model is viewed as acceptable at CFI ≥ 0.90. In addition, Hu and Bentler (1999) suggest that for a good fit, SRMR should not be more than 0.08. Furthermore, RMSEA values ≤0.06 is regarded to be of good fit, RMSEA value ≤0.08 is of fair fit, RMSEA value between 0.08 and 0.10 is considered mediocre fit, and RMSEA value >0.10 is regarded as poor fit (Hu and Bentler, 1999). Table 3 shows the descriptive statistics and associations of the latent factors in vocabulary learning attitudes and self-regulation in EFL vocabulary learning.

**Table 3:** Descriptive statistics and latent inter-correlations of variables.

	M (latent mean)	SD	1	2	3
Vocabulary attitude 1	2.23	0.39			
Vocabulary attitude 2	2.52	0.58	0.697		
Self-regulation 1	3.26	0.31	0.472	0.367	
Self-regulation 2	3.37	0.35	0.192	0.276	0.573

As shown in Table 3, the variables were correlated significantly at different time waves. There were positive, medium-sized, and negative relationships between the variables. However, the relationship between vocabulary learning attitudes at Time Wave 1 and self-regulatory vocabulary learning at Time Wave 2 was not significant.

## Results

### Within-Person Change

According to McArdle and Prindle (2008), factor structure and longitudinal invariance need to be tested prior to checking the latent change score models. Univariate latent change score models of EFL learners’ vocabulary learning attitudes and their self-regulatory vocabulary learning were set up to examine the amount of change in the two variables over time.

In order to investigate vocabulary learning attitude change over time, a latent change score model was set in which the overall fit indices of the model turned out to be acceptable (*χ*^2^/*df* = 1.79, CFI = 0.96, RMSEA = 0.05, *p* < 0.00). In addition, the latent change variable mean was significant [*M* = 0.28 (*SE* = 0.03), *p* < 0.00] revealing that there was a significant increase of vocabulary learning attitudes from Time Wave 1 to Time Wave 2. Furthermore, vocabulary attitudes at Time Wave 1 with sigma = 0.15 (*SE* = 0.02), *p* < 0.00 and the latent change variable with sigma = 0.19 (*SE* = 0.03), *p* < 0.00 indicated a significant value of variance, reflecting individual differences both in vocabulary learning attitudes at Time Wave 1 and the change to Time Wave 2.

Concerning self-regulatory vocabulary learning change over time, the fit indices of the latent change score model for self-regulatory capacity in EFL vocabulary learning was acceptable (*χ*^2^/*df* = 1.68, CFI = 0.94, RMSEA = 0.04, *p* < 0.00). The latent change variable mean was significant [*M* = 0.24 (*SE* = 0.03), *p* < 0.00] indicating an increase of self-regulatory vocabulary learning from Time Wave 1 to Time Wave 2. Moreover, there was a significant variance at Time Wave 1 with sigma = 0.21 (*SE* = 0.04), *p* < 0.00 and also a significant variance in the latent change variable with sigma = 0.19 (*SE* = 0.02), *p* < 0.00. Therefore, it can be suggested that there were significant individual differences within the variables under investigation.

### Between-Person Differences in Change

The two univariate latent change score models were integrated in one bivariate latent curve model to examine the dynamic relationships of EFL learners’ attitudes towards vocabulary learning and their self-regulatory vocabulary learning over time. In this bivariate model, the predictive impact of both variables at a previous stage (i.e., Time Wave 1) in predicting change in the other variable can be tested. Moreover, through the bivariate model, it can be tested if there is a correlation between both change variables. The fit indices of the bivariate model were acceptable (*χ*^2^/*df* = 1.98, CFI = 0.95, RMSEA = 0.04, *p* < 0.00).

Moreover, the relationship of vocabulary learning attitudes and self-regulatory capacity in vocabulary learning at Time Wave 1 was significant and positive (*β* = 0.44 and *p* < 0.00). The association of change variables was also significant and positive (*β* = 0.39 and *p* < 0.00). This means that learners who demonstrated high improvements in vocabulary learning attitudes had also strong improvements in self-regulatory capacity in vocabulary learning. This indicated that high levels of vocabulary learning attitude at Time Wave 1 were related to high levels of self-regulatory capacity increases over time.

## Discussion

Drawing on Engeström’s (1987) activity theory, the current study was set to examine the impact of a MALL programme on EFL learners’ vocabulary learning attitudes and self-regulatory capacity in vocabulary learning through a quantitative longitudinal research design. Two latent change score models and their interrelations were tested in the present study: within-person changes of vocabulary learning attitudes and self-regulatory vocabulary learning and between-person differences (individual differences). First, the results of within-person differences revealed overall increase in both vocabulary learning attitudes and self-regulatory vocabulary learning. In other words, the involvement of learners in MALL activities contributed to positive vocabulary learning attitudes and self-regulatory capacity in vocabulary learning over time.

The results of the present study could be in line with the findings of Ellis (2008), Burston (2015), and Liu (2016) who confirmed the beneficial role of mobile-integrated language learning procedures in creating positive learning attitudes. Rachels and Rockinson-Szapkiw (2018) also argued that mobile-based instruction developed learners’ positive perceptions towards vocabulary learning. The findings of this study seem justifiable since mobile devices are likely to have provided the participants with collaborative activities both in and outside the classroom, thereby, contributing to foster positive learning attitudes among EFL learners. This means that the mobile applications possessed a lot of affordances for involving the learners in collaborative activities so that the learners could carry out the vocabulary tasks more conveniently and enthusiastically. In agreement with the findings, Lu (2008) also claims that MALL encourages interactions among the learners, enhances integrations among natural communication needs and language learning, and fosters retention of language learning skills.

Furthermore, following Engeström’s (1987) activity theory, the researcher/instructor’s and the other peers’ mediations engaged the learners in vocabulary-based activities. The instructor’s and other peers’ mediations along with the MALL environment helped the learners reach their self-regulation. Different applications available in the MALL space helped the learners have more effective and efficient peer mediations on each other’s communicative activities, which could further contributed to their vocabulary improvements. The findings of the present study showed that the MALL programme helped the EFL learners engage in other-regulation activites, following Engeström’s (1987) activity theory, as they were willing to use the MALL applications for vocabulary improvement purposes. The learners’ other regulations in the MALL environment also helped the learners reach their self-regulation in vocabulary development. That is, the learners gradually became autonomous in using the MALL applications appropriately for improving their vocabulary knowledge. This could be regarded as another evidence of the MALL learners’ high vocabulary improvements. The EFL learners also had more positive vocabulary learning attitudes which might further substantiate the above-mentioned findings of the study.

With regard to the improvements in self-regulatory vocabulary learning, the findings of the present study are consistent with those of Shea and Bidjerano (2010) and Lai and Gu (2011) who emphasised the effects of technological devices on fostering self-regulated learning. According to Zimmerman (2000), self-regulated learners are able to become meta-cognitively, motivationally, and behaviourally active participants of their own learning processes. From this perspective, the learners of the present study were meta-cognitively, motivationally, and behaviourally active in the sense that MALL helped them set more appropriate goals and use effective strategies to manage processes and content and to engage in help-seeking, self-monitoring, and self-evaluation. It could be argued that MALL marks a shift from teacher-driven learning to student-driven learning which might help students become autonomous and active learners, take the responsibility of their own learning, and be able to self-regulate their learning processes. In addition, as mobile vocabulary learning applications provide the language learners with the opportunity to “control their learning process, learn at their own pace and learn for their learning needs without being confined in fixed class schedules and physical settings” (Wang and Shih, 2015, p. 373), they enable learners to self-regulate their learning processes.

These findings are also consistent with Engeström’s (1987) activity theory, as learners self-regulation of learning is the desired outcome which they try to achieve following their interactions with other peers. Based on the Engeström’s (1987) activity theory, the MALL applications allowed the more capable peers to use self-regulatory strategies to mediate the less capable learners so that they could increase their vocabulary knowledge in the MALL environment more effectively. The community of more and less capable learners in the MALL environment allowed the less capable learners became more engaged in the communicative tasks as the capable peers were there to contribute to their vocabulary knowledge. These peer mediations between the less and more capable learners in the MALL space allowed both the more and less capable learners improve their vocabulary knowledge, and hence, their in self-regulatory vocabulary learning.

Between-person differences of change were also examined to address the second purpose of the present study. The interrelation of EFL vocabulary learning attitudes and self-regulatory capacity in vocabulary learning was investigated using bivariate change models in which each variable is considered a predictor for the other. The findings in this regard indicated that change in vocabulary learning attitudes and self-regulatory capacity in vocabulary learning was positively correlated. That is, EFL learners who showed high improvements in vocabulary learning attitudes were willing to show more self-regulatory capacity in vocabulary learning, whereas EFL learners who showed less improvements in vocabulary learning attitudes had a tendency to show less self-regulatory capacity in vocabulary learning. The reciprocal relationship between learning attitudes and self-regulated learning was corroborated by Zimmerman and Schunk (2008) and Ning and Downing (2010). These findings demonstrated that learners with positive learning attitudes were more likely to show self-regulatory and achievement-oriented behaviours. Based on activity theory (Engeström, 1987), learners who were actively engaged in vocabulary-based interactions in MALL environment (i.e., other-regulating their vocabulary knowledge) were better able to achieve their self-regulation of vocabulary learning.

The current study employed a dynamic rather than a static procedure to assess changes *via* latent change score models. This procedure helped identify model changes in EFL learners’ vocabulary learning attitudes and learners’ self-regulatory vocabulary learning, as well as their interrelation, allowing the investigation of within-person changes and between-person differences in change. However, the EFL learners’ vocabulary learning attitudes and self-regulatory vocabulary learning were measured through self-report scales. Moreover, since the changes in both variables were assessed simultaneously, causality inference in the relationship of the two variables changes were not possible. Given the fact that the present study utilised a two-wave set of data in the analysis, future EFL researchers are recommended to include more time waves and investigate more detailed analyses of change. Following Ellis (2015), in order to confirm changes in language acquisition, research data need to be based on learners’ social activities or participations and learners’ change in a particular and different context.

## Conclusion

The current study showed the importance of MALL in fostering EFL learners’ vocabulary learning attitudes and self-regulatory capacity in vocabulary learning. With regard to the fruitful results of the present study, implications for EFL learners, teachers, and educators are suggested. For instance, the vital role of MALL in enhancing EFL learners’ vocabulary learning attitudes and regulating their English vocabulary learning were confirmed in the present study, although some learners did not consider MALL efficient in regulating their vocabulary knowledge. The insular learners changed their mind about efficiency of MALL in boosting their vocabulary knowledge, as they found themselves enriched in pervasive domination of vocabulary at the end of the course due to using numerous mobile applications.

Not only language learners, but also EFL teachers can benefit from the findings of the present study. For instance, they may enjoy mobile-based applications to support the teaching process. To this aim, EFL teachers need to be equipped with a pre-packed collection of mobile applications appropriate for theirlanguage teaching and learning context. The results of this study further encourage EFL teachers not to see mobile phones as an interruptive device in EFL education contexts but rather as a great facilitator of English vocabulary learning in classroom. It is also implied that mobile-based applications facilitate both learner-learner and learner-teacher interactions. Çetinkaya and Sütçü (2018), in this regard, argue that mobile-based applications help learners have more in-class and out-of-class interactions. Moreover, Çetinkaya and Sütçü (2018) claim that mobile applications expedite the question-and-answer interplay between learners and teachers. Following the findings of the current study, EFL teacher educators and policy makers might provide and facilitate a convenient condition for EFL teachers and learners to apply such mobile-based applications in their vocabulary learning courses.

Additionally, incorporating mobile-based applications for improving the vocabulary knowledge of EFL learners, enhances both learner-learner and instructor-learners interactions during and outside the class. EFL learners can have and receive more peer- and instructor- vocabulary mediations and improve their vocabulary knowledge in that regard (Rassaei, 2020). Incorporating mobile-based applications can also increase EFL learners’ motivation, which can help them enhance their vocabulary knowledge and self-regulatory vocabulary learning. However, it is important to instruct EFL instructors to incorporate appropriate mobile-based applications in their EFL courses. EFL learners need to be familiarised with the mobile-based applications, so as to apply the applications not only when they are engaged in collaborative activities with their peers, but also when they use the applications autonomousely (when they achieve their self-regulation in vocabulary learning), like those of Rahimi and Fathi (2021) and Rassaei’s (2021) study in which learners achieved their self-regulation after providing and receiving implicit and explicit peer mediations. Following Simpson (2005), getting an acceptable level of digital literacy seems to be a fundamental prerequisite to conduct mobile-based courses in EFL contexts. Digital literacy could be defined as “the ability to use information and communication technologies to find, evaluate, create, and communicate information, requiring both cognitive and technical skills” (Digital Literacy Task Force, 2013, p. 2). Therefore, increasing EFL learners’ digital literacy might further help the learners perform better in mobile-based vocabulary learning environments.

However, the findings of this study need to be generalised with caution as it was conducted among specific participants in EFL setting. Additionally, due to learner differences in cognition and English proficiency level, which could probably affect the way the learners in the present study were engaged in the mobile-based environments, the EFL researchers are recommended to consider more in-depth studies about EFL learners with similar cognition and English proficiency level. It is also worthwhile to propose that neither superiority nor priority of any special kind of mobile phone was taken into consideration during this study. And even the practicality and applicability of mobile phones over other mobile technologies, such as PDAs, iPads, or tablets, were not regarded as the vital emphasis of this study.

Furthermore, as we could not make sure if the participants used the applications consistently and if they did not involve in other modes of learning, other researchers might carry out a similar study and adopt an online programme and instrument to check and record EFL learners’ use of the applications. This could be controlled somehow, but as the nature of the longitudinal study, other factors might be involved affecting learners’ improvement in a long period of time which cannot be controlled completely. Moreover, as we only used one group of learners, other researchers may add a control group of learners who do not use mobile based applications to make a comparison between the experimental and control groups’ vocabulary learning attitudes and self-regulatory capacity in vocabulary learning so that the findings could be generalised and more valid.

In the Iranian context, on the other hand, vocabulary is usually decontextualised and taught as a separate skill. As a result, EFL learners do not usually find opportunities to use the words in their speaking and writing activities. That is, following Li and Hafner (2022), instructors in the EFL contexts only try to enhance EFL learners’ receptive knowledge of vocabulary by teaching vocabularies individually or through listening and reading skills; however, instructors ignore EFL learners productive knowledge of vocabulary. This means that in the EFL contexts, learners are not usually involved in speaking and writing activities to use their vocabulary knowledge productively. As the current study indicated, mobile-based applications provide a user-friendly and convenient online environment for EFL student not only to improve their receptive knowledge of vocabulary, but also to apply their newly acquire vocabularies productively through collaborative activities with their peers.

## Data Availability Statement

The data analyzed in this study is subject to the following licenses/restrictions: the dataset will be available upon request by contacting the corresponding author. Requests to access these datasets should be directed to jfathi13@yahoo.com.

## Ethics Statement

The studies involving human participants were reviewed and approved by University of Kurdistan. The patients/participants provided their written informed consent to participate in this study.

## Author Contributions

All authors have contributed equally to data collection, data analysis, research questions, topic development, writing the manuscript as well as its revision, and language editing.

## Funding

This paper was supported by Blended Learning Design and Practice of EFL Courses based on BOPPPS and an APP Named Chaoxing Learning (ZH202138), A Research on the Teaching Effect Evaluation of Ideological and Political Education in Medical Humanities (2021RWJD01SZ), and A Research on the Teaching Design and Application of Undergraduate Dissertations in English Majors (202102431018).

## Conflict of Interest

The authors declare that the research was conducted in the absence of any commercial or financial relationships that could be construed as a potential conflict of interest.

## Publisher’s Note

All claims expressed in this article are solely those of the authors and do not necessarily represent those of their affiliated organizations, or those of the publisher, the editors and the reviewers. Any product that may be evaluated in this article, or claim that may be made by its manufacturer, is not guaranteed or endorsed by the publisher.
